# Elevated Microsatellite Alterations at Selected Tetranucleotide Repeats (EMAST) in Penile Squamous Cell Carcinoma—No Evidence for a Role in Carcinogenesis

**DOI:** 10.3390/curroncol31100427

**Published:** 2024-09-25

**Authors:** August Fiegl, Olaf Wendler, Johannes Giedl, Nadine T. Gaisa, Georg Richter, Valentina Campean, Maximilian Burger, Femke Simmer, Iris Nagtegaal, Bernd Wullich, Simone Bertz, Arndt Hartmann, Robert Stoehr

**Affiliations:** 1 Institute of Pathology, University Hospital Erlangen-Nürnberg, Friedrich-Alexander-Universität Erlangen-Nürnberg (FAU), 91054 Erlangen, Germany; august.fiegl@uk-erlangen.de (A.F.); johannes.giedl@pathologie-weiden.de (J.G.); simone.bertz@uk-erlangen.de (S.B.); arndt.hartmann@uk-erlangen.de (A.H.); 2 Comprehensive Cancer Center Erlangen-EMN (CCC ER-EMN), 91054 Erlangen, Germany; olaf.wendler@uk-erlangen.de (O.W.);; 3 Bavarian Cancer Research Center (BZKF), 91052 Erlangen, Germany; 4 Department of Otorhinolaryngology—Head and Neck Surgery, University Hospital Erlangen-Nürnberg, Friedrich-Alexander-Universität Erlangen-Nürnberg (FAU), 91054 Erlangen, Germany; 5 Institute of Pathology, University Hospital Ulm, 89081 Ulm, Germany; 6 Institute of Pathology, RWTH Aachen University, 52062 Aachen, Germany; 7 Institute of Pathology, 31785 Hameln, Germany; richter@pathologie-richter.com; 8 Institute of Pathology, 91522 Ansbach, Germany; campean@patho-ansbach.de; 9 St. Josef Medical Centre, Department of Urology, University Regensburg, 93053 Regensburg, Germany; mburger@csj.de; 10 Department of Pathology, Radboud University Medical Centre, 6525 GA Nijmegen, The Netherlands; femke.doubrava-simmer@radboudumc.nl; 11 Department of Urology, University Hospital Erlangen-Nürnberg, Friedrich-Alexander-Universität Erlangen-Nürnberg (FAU), 91054 Erlangen, Germany; iris.nagtegaal@radboudumc.nl

**Keywords:** penile cancer, EMAST, MSH3

## Abstract

Penile squamous cell carcinoma (pSCC) is a rare malignancy with a global incidence ranging from 0.1 to 0.7 per 100,000 males. Prognosis is generally favorable for localized tumors, but metastatic pSCC remains challenging, with low survival rates. The role of novel biomarkers, such as tumor mutational burden (TMB) and microsatellite instability (MSI), in predicting the response to immune checkpoint inhibitors (ICIs) has been investigated in various cancers. However, MSI has not been observed in pSCC, limiting immunotherapy options for this patient subgroup. Elevated microsatellite alterations at selected tetranucleotide repeats (EMAST) are a distinct form of genomic instability associated with deficient MSH3 expression, which has been proposed as a potential biomarker in several cancers. This study investigates EMAST and MSH3 expression in a cohort of 78 pSCC cases using PCR, fragment analysis and immunohistochemistry. For the detection of EMAST, the stability of five microsatellite markers (D9S242, D20S82, MYCL1, D8S321 and D20S85) was analyzed. None of the cases showed an instability. As for MSH3 immunohistochemistry, all analyzable cases showed retained MSH3 expression. These results strongly suggest that neither EMAST nor MSH3 deficiency is involved in the carcinogenesis of pSCC and do not represent reliable predictive biomarkers in this entity. Furthermore, these findings are in full agreement with our previous study showing a very low frequency of MSI and further support the thesis that EMAST and MSI are strongly interconnected forms of genomic instability. Further research is needed to explore novel therapeutic targets and predictive biomarkers for immunotherapy in this patient population.

## 1. Introduction

Penile squamous cell carcinoma (pSCC) is considered a rare malignant tumor, with a global incidence of 0.1 to 0.7 annual cases per 100,000 males. Incidence shows wide geographic variations, with four times higher disease rates in countries like Brazil or Uganda compared to Western Europe [1]. Most pSCCs arise as a result of chronic infection with high-risk types of human papilloma virus (HPV), most commonly HPV-16 and -18. HPV-negative pSCCs usually arise in the background of chronic inflammation, such as penile lichen sclerosus or lichen planus, and show a more aggressive clinical course. For localized tumors, where organ-preserving local excision and/or radiation is sufficient, prognosis is generally favorable, with 5-year overall survival (OS) rates of 79% [2,3]. In spite of aggressive multimodal treatment strategies, such as lymphadenectomy, adjuvant radiation therapy and/or platin-based chemotherapy, metastatic disease involving inguinal lymph nodes or distant organ sites shows a poor 5-year OS of 51% and 9%, respectively [4]. 

Recently, biomarkers related to the tumor neoepitope burden, such as the tumor mutational burden (TMB) or microsatellite instability (MSI), have been shown to predict the response to immune checkpoint inhibitors (ICIs) in various organs, resulting in a tumor-agnostic FDA approval of pembrolizumab for MSI-high (MSI-H) and TMB-high (TMB-H) tumors [5,6]. 

MSI is defined as the accumulation of deletions and insertions in mono- and dinucleotide short tandem repeats (STRs) dispersed across the whole genome and is caused by a deficiency in the mismatch repair (MMR) system. However, the absence of MSI in pSCC found by our group in a large cohort study has limited the role of immunotherapy in this patient group although immune checkpoint inhibitors recently showed promising results in a subset of pSCC patients [7,8]. This issue raises an unmet need to explore novel therapeutic targets and predictive biomarkers for immunotherapy.

In 2000, researchers discovered a distinct form of MSI associated with a deficient expression of the MMR protein MSH3, resulting in instability at tetra-nucleotide repeat motifs (e.g., (AAAG)n or (ATAG)n), termed elevated microsatellite alterations at selected tetranucleotide repeats (EMAST) [9,10,11,12,13]. Succeeding studies observed EMAST in several other tumor entities, including skin, ovarian, colorectal, bladder and endometrial, and identified unique associations with clinicopathological features: colorectal cancer (CRC)-positive for EMAST and MSI demonstrated a better OS, higher prevalence in the proximal colon, predominance in female patients, mucinous differentiation and higher incidence of co-mutations such as PI3KCA, BRAF, PTEN and AKT1 compared with tumors without EMAST [11,14,15,16]. Additionally, EMAST has recently been linked to high levels of CD8+ tumor-infiltrating lymphocytes and PD-L1 expression in tumor and immune cells of CRC, suggesting increased immunogenicity and a potential value as a predictive biomarker for ICI [17]. To the best of our knowledge, no studies have investigated the role of EMAST in pSCC. Therefore, we herein investigate EMAST status using PCR fragment analysis and MSH3 expression using immunohistochemistry in a large cohort of pSCC.

## 2. Materials and Methods

### 2.1. Patients and Tissue Samples

**Figure 1:**
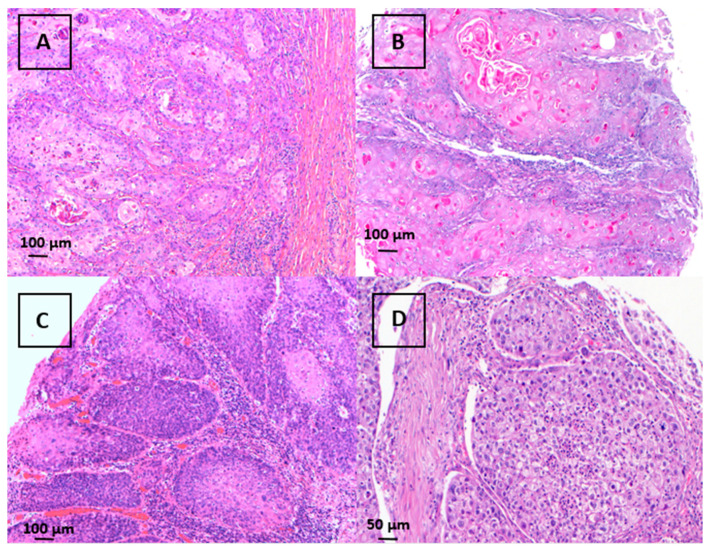
Examples of different histological subtypes of keratinizing and non-keratinizing penile squamous cell carcinomas in H&E ((**A**): usual type, 100×; (**B**): verrucous type, 100×; (**C**): basaloid type, 100×; (**D**): clear cell type, 200×).

This investigation utilized archival formalin-fixed, paraffin-embedded (FFPE) tissue samples obtained from 78 cases of penile SCC, comprising both tumorous and non-tumorous tissue. Patient tumors were categorized and staged in accordance with the WHO classification of penile tumors [18] and the current AJCC/TNM classification system [19]. Given that the current WHO classification of penile SCC incorporates the HPV status of the tumor (HPV-associated versus HPV-independent), all cases included in this study underwent analysis to determine the presence or absence of HPV including subtyping. Detailed clinicopathological characteristics of the cases are presented in Table 1 and Figure 1. 

### 2.2. Microdissection and DNA Isolation

The genomic DNA extraction process from FFPE tissue involved microdissection and isolation, following a previously outlined protocol [20]. Five µm thick consecutive tissue sections were firstly dewaxed, secondly rehydrated and briefly stained with methylene blue (0.1%) for 15 s. An inverted microscope was used to separate and meticulously collect tumorous and non-tumorous tissue from the sections with a sterile needle, with their identification verified by comparison with a labeled H&E-stained section examined by an experienced surgical pathologist. Extracted tumor cells have a confirmed purity of at least 80%. Genomic DNA was isolated from the microdissected tissue using the Blood DNA Preparation Kit (Maxwell^®^ 16 System, Promega, Mannheim, Germany), following the manufacturer’s guidelines.

### 2.3. EMAST Detection

The microsatellite status of the DNA samples derived from tumor cells and corresponding normal tissue was determined by PCR analysis. For EMAST detection, the following consensus markers and primers were used: MYCL1 (sense: 5′-TGG CGA GAC TCC ATC AAA G-3′, antisense: 5′-CCT TTT AAG CTG CAA CAA TTT C-3′); D8S321 (sense: 5′-GAT GAA AGA ATG ATA GAT TAC AG-3′, antisense: 5′-ATC TTC TCA TGC CAT ATC TGC-3′); D9S242 (sense: 5′-GTG AGA GTT CCT TCT GGC-3′, antisense: 5′-ACT CCA GTA CAA GAC TCT G-3′); D20S82 (sense: 5′-GCC TTG ATC ACA CCA CTA CA-3′, antisense: 5′-GTG GTC ACT AAA GTT TCT GCT-3′) and D20S85 (sense: 5′-GAG TAT CCA GAG AGC TAT TA-3′, antisense: 5′-ATT ACA GTG TGA GAC CCT G-3′). Primer sequences were already published before [21]. Approximately 100 ng of DNA was employed for PCR amplifications. The ideal PCR conditions (e.g., annealing temperature) were determined using gradient PCR. Microsatellite PCR was performed using the Multiplex PCR Kit (QIAGEN, Hilden, Germany) and 0.18 µM of each primer (metabion, Planegg, Germany). PCR conditions were as follows: initial denaturation at 95 °C for 2 min followed by 35 cycles of 94 °C for 30 s, 56 °C for 90 s and 72 °C for 60 s, followed by a final elongation step of 72 °C for 30 min. The received amplification products were further analyzed by capillary electrophoresis on an ABI Prism 3500 Genetic Analyzer. For fragment analysis, GeneMapper Software Version 4.1 was used (both Applied Biosystems, Foster City, CA, USA). EMAST was defined as the presence of novel bands or band shifts after PCR amplification of tumor DNA that were not present in the PCR products of the DNA from corresponding normal tissue. A tumor was defined as showing EMAST if at least 2/5 markers analyzed showed instability. In the case of only 1/5 instable markers, the tumor was classified as stable (non-EMAST) [22].

### 2.4. Immunohistochemical Analysiys of MSH3

In order to analyze the MSH3 expression in a tissue-preserving way, a tissue microarray (TMA) was assembled by using single tissue cores (1.2 mm in diameter) taken out from each available paraffin block, following previously published methods, to ensure consistent standards for immunohistochemical analysis [23]. We used immunohistochemistry to analyze the expression of the MMR protein MSH3. The 5 µm sections underwent uniform treatment and staining procedures using the BenchMark ULTRA autostaining system (Ventana Medical Systems, Tucson, AZ, USA) with the iView DAB Detection Kit (Ventana Medical Systems; Tucson, AZ, USA). Protein expression was assessed following standard immunohistochemistry protocols with a specific monoclonal anti-MSH3 antibody (Abcam, clone EPR4334(2), Cambridge, UK, diluted 1:10,000). Evaluation of slides was performed by one surgical pathologist (A.H.) blinded to clinical data. MSH3 protein expression was categorized as negative or positive. Tumors with complete absence or nuclear expression in less than 5% of tumor cells (with regular expression in associated normal tissue) were considered negative. Cases with both strong and weak nuclear positive reactions were classified as positive.

### 2.5. HPV PCR Analysis of Penile Tumors

Detection of HPV in the DNA derived from pSCC tissue was performed in a two-step procedure. In the first step, GP5+/6+ primers selected from the HPV LI region were used for the universal detection of HPV DNA [24]. In the second step, subclassification of HPV species was performed for positive cases using type-specific primers detecting HPV subclasses 11, 16, 18, 31, 33, 35, 39, 45, 52, 53, 58, 59, 66 and 68 as described elsewhere [25,26].

## 3. Results

### 3.1. Detection of HPV in pSCC Cases

The determination of the HPV status was successfully performed in all cases. HPV positivity was found in 28/78 (36%) cases with most of the cases (17/28; 61%) showing known high-risk HPV subtypes (HPV 16, 18, 31, 33, 45, 52 and 58).

### 3.2. Immunohistochemical Evaluation of MSH3 Expression

**Figure 2:**
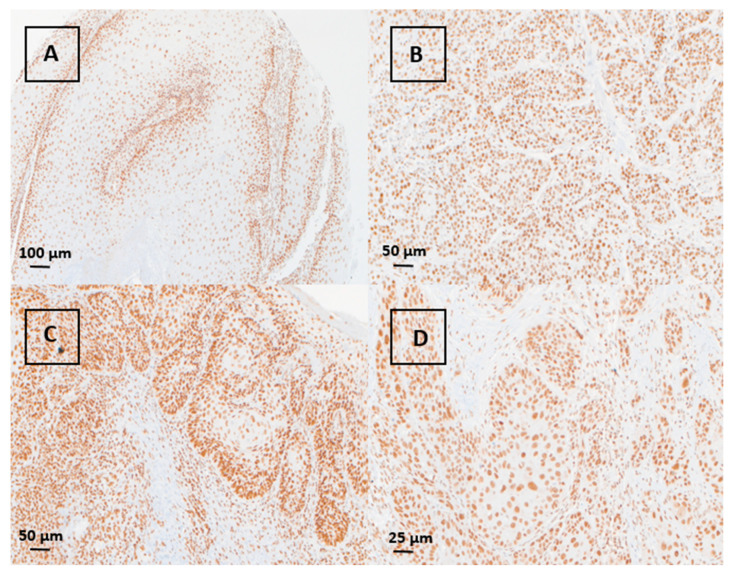
Immunohistochemical analysis of MSH3: retained nuclear MSH3 expression in representative cases of pSCC ((**A**): verrucous type, 40×; (**B**): basaloid type, 100×; (**C**): usual type, 100×; (**D**): usual type, 200×).

Expression of MSH3 in our pSCC cohort was analyzed by immunohistochemical staining of one slide of the TMA. Overall, 55/60 cases on the TMA could be evaluated. The tissue spots of the remaining five cases were lost during the staining procedures. All analyzable pSCC cases showed a retained nuclear MSH3 staining. None of the cases showed a loss of expression (Figure 2). These data also suggest that MSH3 expression might not be related to the HPV status as all analyzable cases on the TMA consisting of both HPV-positive and HPV-negative cases presented strong MSH3 expression. 

### 3.3. Determination of EMAST Status

**Figure 3:**
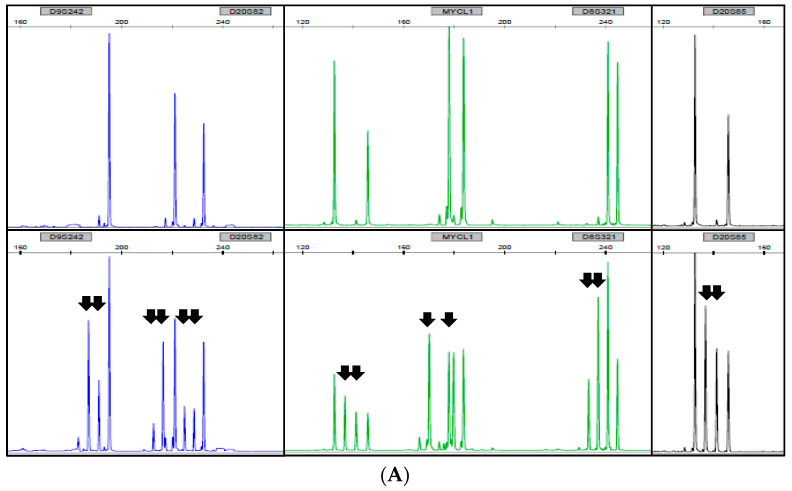

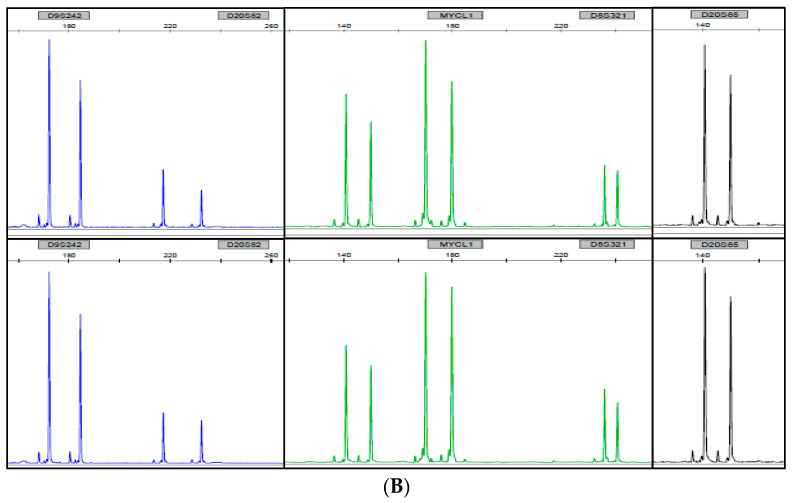
Representative examples for EMAST analysis. (**A**) PCR fragment analysis of an EMAST-positive colon carcinoma (upper lane: normal tissue; lower lane: colon carcinoma). Black arrows indicate additional bands due to microsatellite instability in the DNA from colon carcinoma. This DNA from a previously proven EMAST-positive colon carcinoma served as positive control for our analyses [21]. (**B**) PCR fragment analysis of a pSCC sample. No additional bands are visible in the DNA from pSCC (lower lane) compared to DNA from non-tumoric tissue (upper lane). The shown case was classified as EMAST-negative (stable).

The EMAST analysis of the pSCC cases gave interpretable results in 74/78 (95%) tumors. In 4/78 cases, none of the five EMAST markers investigated could be amplified, indicating a poor DNA quality in these cases. Within the group of pSCC with interpretable EMAST results (n = 74), in 68/74 (92%) tumors, all 5/5 EMAST markers were evaluable, while in 6/74 (8%) cases, only 3/5 EMAST markers were analyzable. Overall, all cases with an evaluable-determined EMAST status showed a stable amplification pattern; EMAST was not detected in any of the cases from our cohort (Figure 3).

## 4. Discussion

The high incidence of EMAST in different cancer types, its unique clinicopathologic features in some entities and strong overlaps with MSI in others raise the need for a better understanding of this form of genomic instability and delineation from MSI in further types of cancer. This seems particularly important given that EMAST might be a promising biomarker for neoepitope burden and could therefore serve as a predictive marker for response to immunotherapy [17]. 

Similar to MSI, the incidence of EMAST varies strongly across different tumor entities: while the highest incidence rates have been observed in colorectal and endometrial cancer (up to 60% and 38,5%, respectively), it is only rarely observed in ovarian, prostate and renal cancer (0–12%, 5% and <1–12%, respectively) [27,28]. One of the unsolved questions about EMAST is whether it represents a clinically distinct form of genomic instability or merely MSI at a more advanced stage caused by additional defects in MMR proteins [29].

In favor of the first theory, no significant association between the incidence of EMAST and MSI was found in a study comprising 65 cases of non-small-cell lung cancer (NSCLC) [30]. The authors identified EMAST, defined as at least 1 instable marker in a panel of 10, in 42 cases (64.6%). Cases of EMAST were further subcategorized as EMAST-high if at least two markers were instable (33.8%). Interestingly, patients with EMAST-high tumors showed poorer overall survival (1394 days versus 2396 days, respectively; *p*-value: 0.0018) and had a higher incidence of additional malignancies, such as gastric or renal cancer, than patients with EMAST-low tumors, irrespective of MSI status. Another study revealed that MSI and EMAST occur independently in urinary tract tumors, by demonstrating a higher prevalence of EMAST in bladder cancer than in the upper urinary tract. The reverse could be demonstrated for MSI, which is more prevalent in tumors of the upper urinary tract than in the bladder [31]. These findings underscore the clinical relevance of EMAST independently of MSI and its potential use as a predisposing factor for the development of multiple primary neoplasms [30].

In favor of the second theory, Ming-Huang Chen et al. evaluated 1505 patients with CRC for the presence of EMAST and MSI, concluding that the majority of cases harboring EMAST demonstrated MSI-H status. In cases exhibiting both EMAST and MSI-H, clinicopathological features commonly associated with MSI-H tumors, such as mucinous differentiation, proximal tumor location, predominance in female patients, lower tumor stage and improved cancer specific survival, were markedly more pronounced than in tumors with MSS/EMAST(+), MSS/EMAST(−) or MSI-H/EMAST(−) profiles. Furthermore, the mutation frequency in MSH-6, MSH-3, PMS2 and EXO1 was higher in cases with EMAST and MSI-H compared to cases only showing EMAST [11].

Taking these findings together, the clinical relevance and prognostic value of EMAST and its association with MSI varies significantly among different tumor entities and should therefore be investigated in tumors that have not been studied for EMAST, such as gliomas or breast cancer. Our study, which analyzed 78 patients with pSCC, found no evidence of EMAST. These data, which are in line with our previous study that found no evidence of MSI in the same patient cohort, provide solid evidence that neither EMAST nor MSI play a role in the development of pSCC [7]. In addition, in our previous study, we also analyzed the expression of the mismatch repair (MMR) proteins MLH1, PMS2, MSH2 and MSH6. We also found no loss of MMR protein expression in any of the analyzed cases. As the analyzed cases partly overlap in both studies, our data further suggest that MMR protein loss is not an important mechanism in pSCC development. 

However, our study certainly has some limitations: Firstly, to date, no validated consensus on a standardized detection panel for EMAST has been established, leading to a limited comparability of studies using different markers and cut-off values. Early studies defined a cut-off of at least two instable markers out of seven (MYCL1, D20S82, D20S85, L17835, D8S321, D9S242 and D19S394) to define EMAST, resulting in prevalence rates as high as 60% in colorectal cancer. However, we decided to define EMAST using a cut-off of two out of five instable markers, aligning with most other studies. This approach resulted in better correlations with clinically relevant features, such as advanced tumor stage and chronic tumor inflammation [22,32]. Although no case in our cohort demonstrated instabilities in any marker, we cannot entirely exclude the possibility of detecting EMAST in certain cases of pSCC when using a larger panel. Nevertheless, our data strongly argue against a significant role of EMAST in the carcinogenesis of pSCC.

As a second limitation, our study is constrained by the racial homogeneity of our patient cohort, primarily consisting of individuals of Caucasian descent. In rectal cancer, for instance, prevalence of EMAST is significantly higher in African Americans compared with Caucasians (49% vs. 26%, *p* = 0.014) [22]. Therefore, our results are representative for Caucasians but do not rule out the possibility that EMAST may exist in pSCC patients of different ethnic backgrounds.

Finally, to explore whether the function of MSH3 is compromised through cytosolic translocation or pathogenic inactivation, we deployed immunohistochemistry to assess the spatial distribution of MSH3 in 55 cases of our cohort. As all cases showed a retained nuclear MSH3 expression, we conclude that MSH-3 inactivation does not commonly occur in pSCC, which aligns with the complete absence of EMAST.

To the best of our knowledge, this is the first study investigating EMAST and the role of MSH-3 in pSCC: all cases were stable in the predefined microsatellite loci and demonstrated intact MSH-3 expression. In summary, these findings demonstrate that EMAST is not involved in the carcinogenesis of pSCC and therefore of no use as a diagnostic or therapeutic biomarker in this entity. 

## Figures and Tables

**Table 1:** Characteristics of the analyzed pSCC cohort.

	Cases (n = 78)	
Age, years		
Median age	68	
Mean age	67.6 ± 11.6	
Range	39–93	
Tumor Stage	n=	
pTis	7	
pT1a	30	
pT1b	5	
pT2	23	
pT3	11	
pT4	1	
unknown	1	
Tumor Grade	n=	
1	16	
2	38	
3	16	
Unknown	1	
HPV Status	n=	
Positive	28	
Negative	50	
Histological subtype	HPV negative (n)	HPV positive (n)
Usual type	31	3
Verrucous	9	-
Basaloid	3	11
Warty-basaloid	1	6
Pseudohyperplastic	3	-
Warty	1	1
Lymphoepithelioma-like	-	1
Clear cell	-	1
Carcinoma cuniculatum	1	-
Unknown: n = 1		

## Data Availability

Dataset available on reasonable request from the corresponding author.
